# Poly(Neopentyl Glycol Furanoate): A Member of the Furan-Based Polyester Family with Smart Barrier Performances for Sustainable Food Packaging Applications

**DOI:** 10.3390/ma10091028

**Published:** 2017-09-04

**Authors:** Laura Genovese, Nadia Lotti, Valentina Siracusa, Andrea Munari

**Affiliations:** 1 Civil, Chemical, Environmental and Materials Engineering Department via Terracini 28, 40131 Bologna, Italy; laura.genovese@unibo.it (L.G.); andrea.munari@unibo.it (A.M.); 2 Department of Chemical Science, University of Catania, Viale A. Doria 6, 95125 Catania (CT), Italy; vsiracus@dmfci.unict.it

**Keywords:** 2,5-furandicarboxylic acid, neopentyl glycol, thermal properties, mechanical properties, barrier properties

## Abstract

In the last decade, there has been an increased interest from the food packaging industry toward the development and application of bioplastics, to contribute to the sustainable economy and to reduce the huge environmental problem afflicting the planet. In the present work, we focus on a new furan-based polyester, poly(neopentyl glycol 2,5-furanoate) (PNF) to be used for sustainable food packaging applications. The aromatic polyester was successfully synthesized with high molecular weight, through a solvent-free process, starting directly from 2,5-furandicarboxylic acid. PNF was revealed to be a material with good thermal stability, characterized by a higher T_g_ and T_m_ and a lower RAF fraction compared to poly(propylene 2,5-furanoate) (PPF), ascribable to the two methyl side groups present in PNF glycol-sub-unit. PNF’s mechanical characteristics, i.e., very high elastic modulus and brittle fracture, were found to be similar to those of PPF and PEF. Barrier properties to different gases, temperatures and relative humidity were evaluated. From the results obtained, PNF was showed to be a material with very smart barrier performances, significantly superior with respect to PEF’s ones. Lastly, PNF’s permeability behavior did not appreciably change after contact with food simulants, whereas it got worse with increasing RH, due to the polar nature of furan ring.

## 1. Introduction

Synthetic plastics are inexpensive, lightweight and durable materials, easily processed into a variety of products that find use in a wide range of applications. Consequently, the production of plastics has increased markedly over the last 60 years [1]. Today, plastics are almost completely derived from petrochemicals, produced from fossil oil and gas. Around 4% of world oil and gas production, non-renewable resources, are used as feedstock for plastics and a further 3–4% is expended to provide energy for their manufacture. Approximately 50% of plastics produced each year are used to make disposable items, such as packaging or other short-lived products that are discarded within a year of manufacture. These two observations alone indicate that current use of plastics is not sustainable. In addition, because of the durability of the polymers involved, substantial quantities of discarded end-of-life plastics are accumulating as debris in landfills and in natural habitats worldwide, generating huge terrestrial as well as marine environmental problems. Recycling is clearly a valid waste-management strategy, reducing environmental impact and resource depletion. However, in case of food contact packaging, it is not very desirable and economically advantageous.

On this ground, bioplastics, i.e., plastics obtained from renewable resources and/or biodegradable, may represent a solution to these urgent needs. The use of bioplastics reduces the dependence on fossil resources, reduces greenhouse gas (GHG) emissions, creates renewable energy and increases the resource efficiency [2,3]. Part of the volumes of bioplastics produced nowadays is moreover recycled alongside their conventional counterparts (e.g., bio-based PE in the PE-stream or bio-based PET in the PET stream), contributing themselves to a more efficient waste management.

Among the different renewable starting materials that have been used for the preparation of bioplastics, furan-based monomers have attracted considerable attention, the most important example being represented by 2,5-furandicarboxylic acid (2,5-FDCA). Its success is mostly due to its use for the synthesis of poly(ethylene 2,5-furanoate) (PEF), currently considered the most credible bio-based alternative to poly(ethylene terephthalate) (PET), thanks to its very interesting physic/mechanical and barrier properties. In fact, PEF displays improved barrier performances and more attractive thermal and mechanical properties than PET. In particular, it is characterized by a higher T_g_ (85 °C vs. 76 °C), a lower T_m_ (211 °C vs. 247 °C) [4], a 1.6 times higher Young’s modulus [4], 11 times lower oxygen permeability [5], 19 times lower carbon dioxide permeability [6] and a 5 times lower water diffusion coefficient [7]. Lastly, the production of PEF would decrease the non-renewable energy use of about 40–50% and the greenhouse gas emissions of 45–55% ca. with respect to PET [8].

Currently, the academic research interest have been also extended to other 2,5-furan dicarboxylate-based polymers, which have been obtained by using aliphatic diols with different length, sugar diols like isosorbide, benzylic structures like 1,4-bishydroxymethyl benzene, and bisphenols like hydroquinone, etc. [9]. Soccio et al., Vannini et al., and Guidotti et al., investigated the barrier properties of these new 2,5-FDCA-based polyesters, of course comparing them with those of PEF [10,11,12].

Recently, Tsanaktsis et al. [13] successfully synthesized poly(neopentyl glycol 2,5-furanoate) (PNF) by melt polycondensation, starting from dimethyl 2,5-furanoate. The new bio-based polyester was mainly subjected to a deep thermal characterization, including melt isothermal crystallization studies.

In the present work, we synthesize PNF starting directly from 2,5-furandicarboxylic acid, and besides the basic physical chemical characterization, mechanical as well as barrier properties of PNF compression molded films were investigated at different temperatures and relative humidity, and correlated to the chemical structure. The functional properties have been then compared to those of both poly(propylene 2,5-furanoate) (PPF), previously prepared in our laboratories, and poly(ethylene 2,5-furanoate) (PEF). Lastly, the permeability behavior after contact with food has been investigated, too.

## 2. Results and Discussion

### 2.1. Molecular Characterization

**Figure 1:**
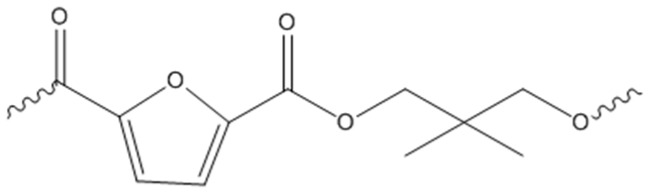
Poly(neopentyl glycol 2,5-furanoate) (PNF) chemical structure.

The chemical structure of the synthesized polymer is shown in Figure 1. The as-prepared sample appeared as a yellowish hard solid material, while the purified one as white floccules.

**Figure 2:**
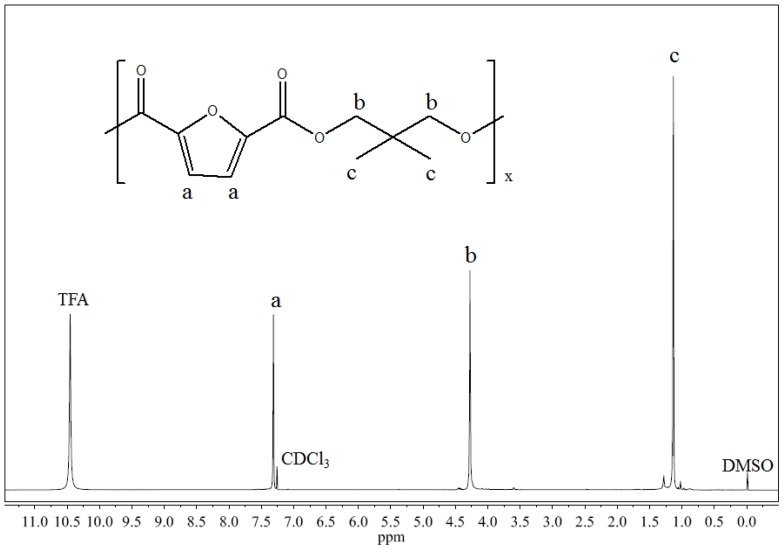
^1^H-NMR spectrum of poly(neopentyl glycol 2,5-furanoate).

^1^H-NMR analysis confirmed the expected structure (Figure 2) and no impurities have been found in the spectrum. In fact, only the peaks due to the polymer were detected: in particular, the chemical-shift assignments (d, ppm) were δ 7.33 (s, 2 Ha), δ 4.27 (s, 4 Hb), δ 1.15 (s, 6 Hc). The polymer is characterized by high molecular weight, very similar to that of PPF previously synthesized by us [12], even though its polydispersity index is larger. Moreover, it should be noted that, unlike Tsanaktsis et al. [13], furan-dicarboxylic acid was directly employed in the synthesis, avoiding the esterification reaction.

The synthesized polyester has been then filmed by compression molding and rapidly cooled in ice water. PPF previously prepared by us [12] was processed into thin film too, and subjected to the same thermal history. As shown by the WCA data reported in Table 1, PNF film appeared to be less hydrophilic than PPF one; this result could be ascribed to the presence of the two side methyl groups in PNF glycol sub-unit.

### 2.2. Thermal Characterization

Afterwards the films have been subjected to thermogravimetric analysis (TGA) under nitrogen flux. The temperatures relative to the degradation onset T_onset_ and to the maximum weight loss rate T_max_ have been reported in Table 1: PNF displayed good thermal stability; thermal degradation began at 364 °C, with the fastest rate at 395 °C.

Degradation completed in one step, and a residual mass of 5% was detected at 750 °C. Similarly to Tsanaktsis et al. [13], T_onset,PNF_ ≅ T_onset,PPF_, whereas T_max,PNF_ > T_max,PPF_. This trend, already observed in other aliphatic and aromatic polyesters [13,14,15], can be ascribed as due to the absence of β-hydrogens in PNF, which are necessary for the β-scission at the ester linkage occurs.

**Figure 3:**
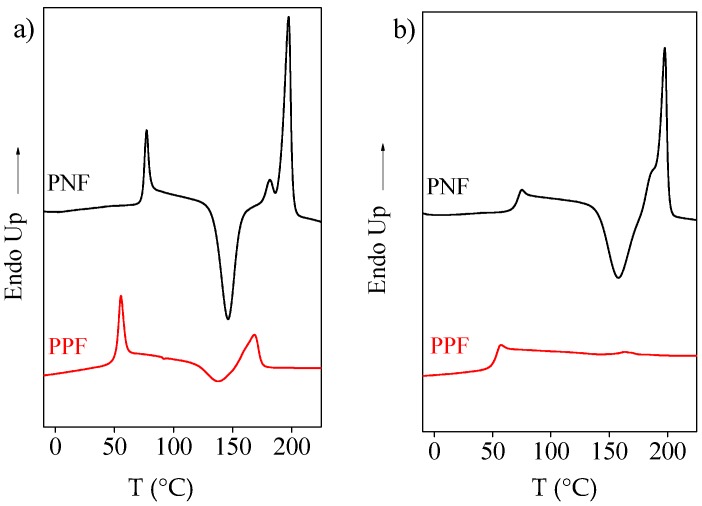
Calorimetric traces of PNF and PPF (20 °C/min): (**a**) 1st scan; (**b**) 2nd scan after melt quenching.

The main thermal transition data of the sample under study are reported in Table 1, together with those of PPF added for sake of comparison [12]. From the data reported in Table 1 and from the DSC curves of Figure 3a, one can see that the two homopolymers display identical phase behavior: both are completely amorphous samples, even though able to crystallize during heating scan once T_g_ is exceeded.

In fact, ∆H_cc_ ≅ ∆H_m_. As to the glass transition phenomenon, both polyesters are characterized by a significant shrinkage, due to processing conditions: as expected, T_g,PNF_ > T_g,PPF_ because of the presence in PNF glycol sub-unit of two side methyl groups in place of hydrogen atoms, which significantly reduce chain flexibility [13,14,15]. PNF is characterized by a T_m_ around 30 °C higher and by a greater crystallizing capacity with respect to PPF. Moreover, PNF DSC trace shows a double melting peak, due to the typical fusion-recrystallization-fusion processes characteristic of polyesters. The higher melting temperature of PNF can be explained taking into account that generally, you should expect that polymers with high T_g_ will also have high T_m_, as the entropy change associated with melting is smaller.

As far as the higher PNF crystallizing ability is concerned, it can be related to the short polymer chains present in this polymer (as confirmed by the large polydispersity index), which could act as self-nuclei. After melt quenching, both polymers keep amorphous, even if PNF still able to crystallize during heating scan, differently from PPF, whose DSC trace appeared to be characterized only by the endothermal baseline deviation due to glass transition phenomenon.

#### Rigid-Amorphous Phase

As recently reported by Tsanaktsis et al. and Soccio et al. [16,17], some semicrystalline furan-based polyesters, such as PEF and PBF, cannot be adequately described by the two-phase model composed of amorphous and crystalline phases. A third phase, called RAF, due to a restricted amorphous phase, frozen by the neighbor crystalline domains, has to be invoked. Both crystalline phase and immobilized amorphous phase contribute to enhance mechanical, gas barrier and other properties of the material.

In order to evaluate the existence of a rigid-amorphous phase in the polymer under investigation and in PPF, the relationship between the specific heat increment at T_g_ and the heat of fusion of samples with different crystal/amorphous ratio was examined (see DSC treatment described in the experimental section). For this purpose, semicrystalline PNF and PPF samples have been obtained by subjecting the corresponding powders to solvent-treatment, which, as well known, favors polymer crystallization.

**Figure 4:**
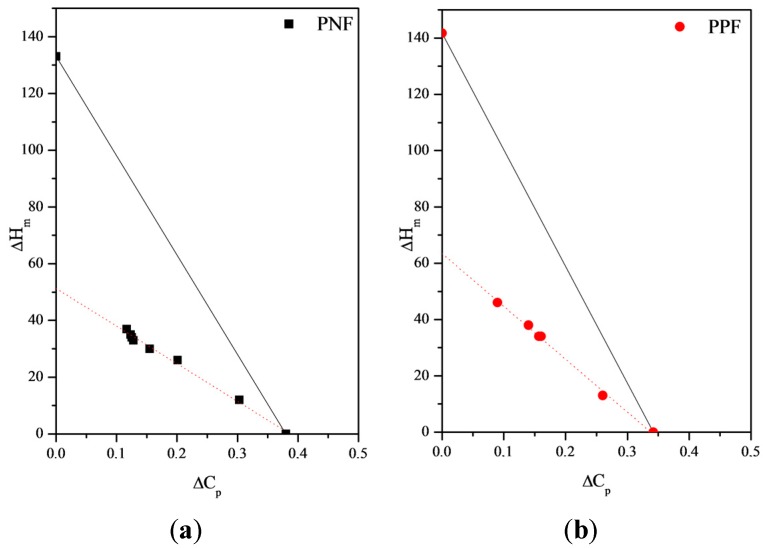
Heat of fusion ∆H_m_ as a function of the specific heat increment ∆C_p_ at T_g_: (**a**) PNF; (**b**) PPF. The solid line was calculated on the basis of the two-phase model.

Figure 4 shows the heat of fusion ∆H_m_ as a function of the specific heat increment ∆C_p_ for these samples; the solid line was calculated on the basis of a two-phase model, considering the equilibrium melting enthalpy of PNF and PPF (133 J/g and 142 J/g, respectively) proposed by Papageorgiou et al. [13,18], and the measured specific heat increment of the completely amorphous samples. As it can be seen, the specific heat increment decreases regularly as the melting enthalpy increases and the data show a very good linear fit. More important, it is clear that the two-phase model is not satisfied for both polyesters, since the experimental specific heat increments of semi-crystalline samples are considerably lower than expected for the full mobilization of the non-crystalline fraction.

The extrapolation to ∆C_p_ = 0 gave values of 51 J/g and 64 J/g for PNF and PPF, respectively, which are significantly lower than the corresponding ΔH_m_° values reported in the literature by Papageorgiou et al. [13,18]; the RAF fraction turned out to be 38% and 45% for PNF and PPF, respectively. In our opinion, the lower RAF fraction found for PNF can be explained as due to its higher crystallizing ability, which gives rise to a crystalline phase characterized by larger spherulites, which exert few constraints on the neighbor amorphous phase.

### 2.3. Permeability Behavior

#### 2.3.1. Barrier Properties

The barrier properties were analyzed by means of permeation measurements to carbon dioxide, oxygen and nitrogen molecules, respectively (CO_2_, O_2_ and N_2_). Such molecules were chosen because are the main gases used for food packaging application, especially for modified atmosphere packaging technique (MAP). In addition, taking into consideration that temperature is one of the most important parameters both for food respiration rate and for polymer gas permeability behavior [19], the barrier properties were evaluated in the range of 8–38 °C, considering all possible temperature scenario from food preservation to food handling. The permeability described as Gas Transmission Rate (GTR) is expressed in cm^3^/m^2^ d bar, as obtained from the instrument. To convert this unit to others reported in literature, the factors reported from Robertson could be used [19].

**Figure 5:**
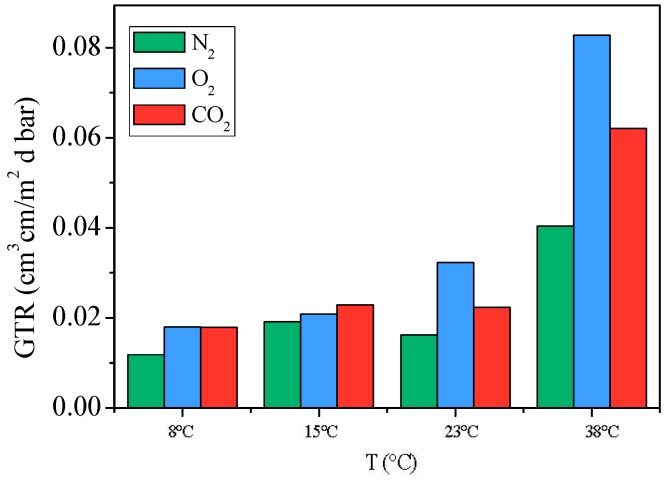
GTR values at the different temperatures for N_2_, O_2_, CO_2_ gases.

In Table 2 are reported the GTR values, normalized for the sample thickness (199 micron), together with the corresponding perm-selectivity ratios and the gas transmission activation energy for N_2_, O_2_ and CO_2_ gases. The GTR values at the different temperatures for the three gases are also plotted in Figure 5.

As evidenced by the data reported in Table 2 and shown in Figure 5, PNF is characterized by very smart barrier properties, despite its amorphous nature. As already reported for PEF by Burgess et al. [5,6,7], such excellent performances can be explained as due to polar furan ring, that, because of nonlinear axis of ring rotation, cannot ring-flip, thus preventing permeant diffusion. Moreover, at 8 and 15 °C GTR_CO_2__ ≅ GTR_O_2__ whereas, at 23 °C and 38 °C, similarly to PEF, GTR value to CO_2_ is lower than GTR value to O_2_ because of CO_2_ higher penetrant sorption and lower diffusion coefficient, due to its polar nature and to the polar furan moiety, which guarantee high level of affinity between gas molecules and polymer matrix. In addition, the presence of the two pendants –CH_3_ groups reduce further the mobility of the polar carbonyl moieties. Lastly, the polymer film showed the best performances with respect to N_2_ gas, despite its non-polar nature, probably because of small molecule dimensions.

A further evidence of PNF smart permeability performances is provided by the Barrier Improvement Factor (BIF) values, reported for oxygen and carbon dioxide in Table 3. The barrier properties of PNF homopolymer film were compared with those of several films reported in literature [5,6,11,12,20,21]. GTR values reported in Table 3 are expressed in cm^3^cm m^−2^ day^−1^ atm^−1^ for sake of comparison. Factors used for converting the permeability from various units are those reported from Robertson [19].

From the data reported in Table 3, it is immediately evident PNF film has very smart barrier performances, competitive with respect to several other polymers. The first comparison has been made on respect to PET, which dominates the market of beverage packaging. As previously done by Burgess [6], the comparison can be effectively quantified in terms of BIF, obtained by dividing the oxygen and carbon dioxide permeability of PET (the sample analyzed at 23 °C was chosen for comparison) with the oxygen and carbon dioxide permeability, respectively of PNF, PPF, PEF and PLA. From permeability data and BIF values reported in Table 3, it is interesting to note as PNF showed the highest BIF values, confirming its superior barrier properties with respect to the polymers taken into consideration in the comparison. In particular, with respect to PET sample, the permeability to CO_2_ and O_2_ gas test is 61 times and 11 times lower, respectively. While these comparisons are far from being exhaustive, they provide meaningful evidence to highlight the potentiality of PNF to be used as high barrier films, especially when high barrier performance against CO_2_ is required, such as for example for carbonated beverages.

#### 2.3.2. Activation Energy of Gas Transport Process

In regions without any transition in polymers and in permeants, the dependence of permeation from temperature can be described through Arrhenius model [19,22]. A linear correlation between a transport parameter logarithm and the reciprocal of the absolute temperature exists:

P = P_0_exp (−E_p_/RT) (1)

where, P is the gas permeability (GTR), P_0_ is a pre-exponential factor of permeation, E_p_ is the activation energy for permeation and R is the gas constant [19,22].

**Figure 6:**
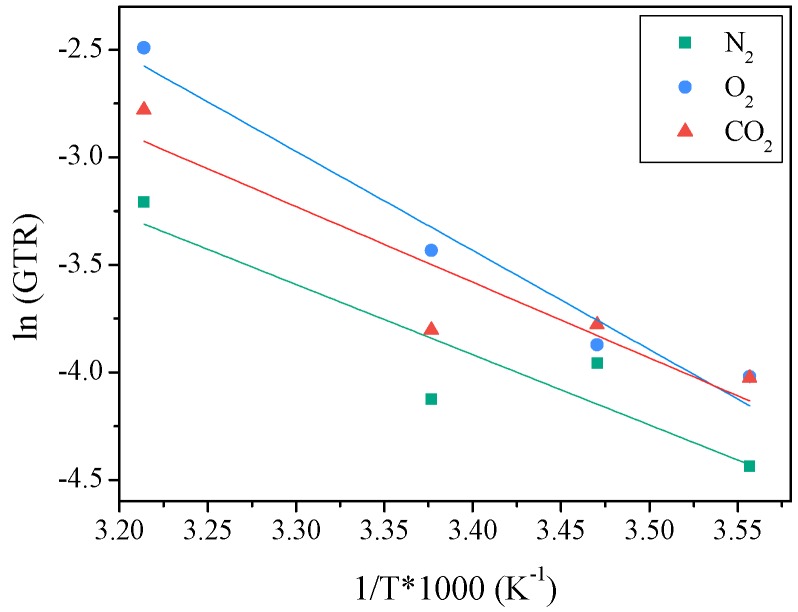
GTR_CO_2__, GTR_O_2__ and GTR_N_2__ values as a function of 1/T (K) for PNF film samples.

Figure 6 reports the GTR dependence of the studied gases from the temperature according to Equation (1). From the linear fitting of the experimental data (solid lines) the activation energies have been calculated and reported in Table 2. Experimental data well fit the theoretical behavior, thus indicating a good correlation between permeability and temperature for all gases.

As is well known from the literature [23], high activation energy implies more sensitivity to temperature variations and therefore the higher the activation energy the higher the GTR variations to temperature changes.

In general, as previously described [24], the activation energy values for gases migrating through a polymeric film range from 12 kJ/mol to 63 kJ/mol. The values calculated for PNF were comprised between 27 kJ/mol and 38 kJ/mol, the highest one being that for O_2_ gas test. Such result confirmed O_2_ molecules move faster than the other gas molecules investigated. Very similar values were reported from Shmid [22] for PET amorphous film. Burgess and collaborators [5,6] reported a value of about 25 KJ/mol for O_2_ gas test and 24 KJ/mol for CO_2_ gas test, for PEF sample, analyzed at 35 °C, in agreement with our data. Recently, we found for PPF sample values of 23 KJ/mol for O_2_ gas test and 30 KJ/mol for CO_2_ gas test. The slightly lower activation energies calculated for PPF could be due to the absence in this polyester of the two side methyl groups in glycol sub-unit.

As reported by Shmid [22], the perm-selectivity describes the permeability ratio between different gases. In general, the ratio of N_2_:O_2_:CO_2_ permeability is in the range of 1:4:16 but, taking into consideration that it is correlated to several parameters, such as chemical structure, gas type and temperature, it could be different, as in our case. In particular, the lower CO_2_/O_2_ perm-selectivity for PNF is in line with the lower E_P,CO_2__ with respect to E_P,O_2__. The perm-selectivity CO_2_/N_2_ was on the contrary nearly constant with T, in agreement with the similar activation energies for the two gases.

#### 2.3.3. Gas Barrier Behavior at Different Relative Humidity

**Figure 7:**
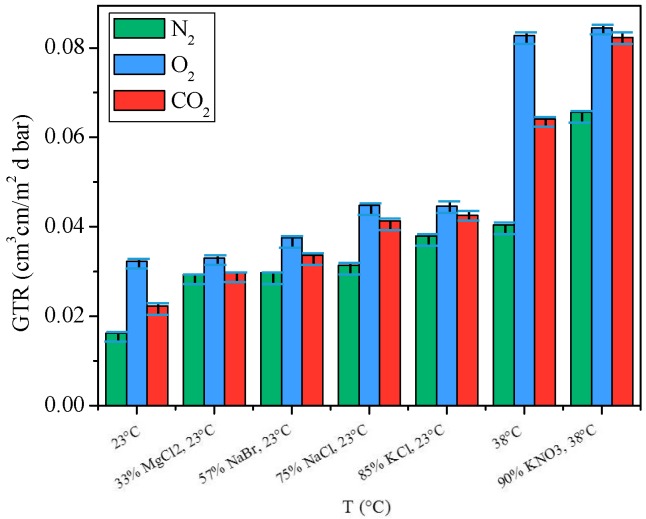
GTR_CO_2__, GTR_O_2__ and GTR_N_2__ values for PNF film samples, under different relative humidity.

The GTR values of PNF films stored at different relative humidity are reported in Figure 7.

As described by Abenojar et al. [25], plasticization and swelling phenomena could occur owing to hydrogen bonds and/or dipole-dipole interactions between the polar polymer chains and water molecules. In particular, according to Meiser et al. [26] and to Lawton et al. [27], the water plasticizing effect causes the loss of small network fragments, promoting the gas transfer throughout the film. The effect of these processes becomes more significant as the percentage of relative humidity and temperature increase.

As shown by Figure 7, a progressive increase in gas transmission rate was recorded at increasing RH, as presumable given the polar character of furan ring. In particular,

- for N_2_ an increment of 82% was recorded at 23 °C from 0% of RH to 85% of RH and of 63% at 38 °C from 0% RH to 90% RH;
- for O_2_ gas an increment of 2.5% was recorded at 23 °C from 0% of RH to 85% of RH and of 2% at 38 °C from 0% RH to 90% RH;
- for CO_2_ an increment of 33% was recorded at 23 °C from 0% of RH to 85% of RH and of 29% at 38 °C from 0% RH to 90% RH.

As can be observed, PNF’s barrier properties got worse at higher relative humidity, highlighting how the water played an important role in the transport process in wet polymer membranes. The wet PNF’s permeability followed the same trend of the dry sample. In fact, wet PNF film showed the highest permeation rate to CO_2_: this result can be explained as due to the higher solubility of this gas caused to strong CO_2_-water interactions [28].

#### 2.3.4. Gas Barrier Behavior after Food Simulants Contact

When polymer films are used for food packaging application, it has to be taken into consideration that polymer film will be in contact with food, and in particular with different kind of food, such as aqueous food, acid food, aqueous food containing oil/fat, oily or fatty food, alcoholic food and low moisture content solid food [29].

In this view, the samples were placed in contact with the food simulant, under the worst of the foreseeable conditions of use as regard contact time and temperature (see Table 1, Table 2 and Table 3 of the EU Regulations) [30]. In particular, test number OM2 was chosen for the experiments to analyze a broad spectra of food packaging scenario with a contact time of 10 days at 40 °C, for any long term food storage at room temperature or below, including heating up to 70 °C for up 2 h, or heating up to 100 °C for up to 15 min. Test OM2 covers also food contact conditions described for OM1 and OM3. As reported from the law, food simulants A, B and C are used for simulating the contact with food characterized by a hydrolitic character and that are able to extract hydrophilic substances. In particular, food simulant B is used for food with pH below 4.5, food simulant C for alcoholic food with an alcohol content up to 20% and those foods containing a relevant amount of organic ingredients that render the food more lipophilic. Food simulant D1 is used for foods that have a lipophilic character and are able to extract lipophilic substances and mimics alcoholic foods with an alcohol content above 20% and oil in water emulsions [30].

**Figure 8:**
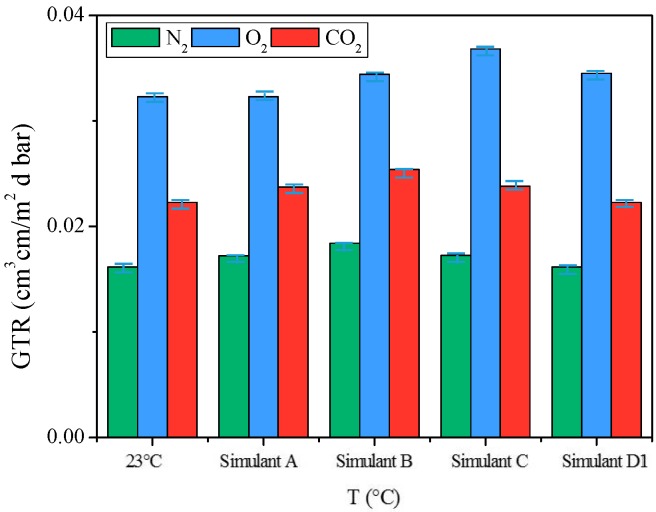
GTR_CO_2__, GTR_O_2__ and GTR_N_2__ values for PNF film samples, after food simulant contact.

As can be observed from the results reported in Figure 8, the highest increment of GTR value was recorded when PNF was in contact with Simulants B, in case of CO_2_ as gas test and with Simulant C when O_2_ was used as gas test, respectively. However, it is worth noticing that in both cases the increment of the GTR are modest, indicating the smart stability of the material when in contact with the food simulants, under the worst condition.

### 2.4. Mechanical Characterization

In an application perspective, the analysis of the mechanical properties is of primary importance. Therefore, the synthesized polymer was subjected to stress-strain measurements (see Experimental part for the measurement conditions). The data (elastic modulus E, stress at break σ_b_, and elongation at break ε_b_) are collected in Table 1: E_PNF_ and σ_b,PNF_ are respectively 17% and 30% higher, than those of PPF, whereas ε_b_ is very similar, despite both polymer films are amorphous [12]. Such mechanical behavior for the two polymers can be explained taking into account that tensile tests have been carried out at room temperature, therefore below their T_g_. Both polymers are therefore in the glassy state. The higher rigidity of PNF can be correlated to its higher glass transition temperature, due to the reduced polymer chain flexibility because of the two side methyl groups in glycol sub-unit [13,14,15]. Similar characteristics, i.e., very high elastic modulus and brittle fracture, have been highlighted for PEF, too [31,32].

## 3. Materials and Methods

### 3.1. Materials

2,5-furandicarboxylic acid (2,5-FDCA) 98% was purchased from CHEMOS GmbH & Co. K (Regenstauf, Germany), whereas, neopentyl glycol (NPG), titanium tetrabutoxide (Ti(OBu)_4_) and titanium tetraisopropoxide (Ti(O-i-Pr)_4_) from Aldrich (Milan, Italy). FDA, NPG and Ti(O-i-Pr)_4_ were used as supplied, whereas Ti(OBu)_4_ was distilled before use.

### 3.2. Polymer Synthesis

Poly(neopentyl 2,5-furanoate) (PNF) was synthesized in bulk too, starting from 2,5-furan dicarboxylic acid (FDCA) and neopentyl glycol (NPG), using a large excess of NPG (500%) with respect to the acid molar content. Antimony trioxide (Sb_2_O_5_) was employed as catalyst (about 7.5 × 10^−4^ mol). The synthesis was carried out in a 250 mL stirred glass reactor, with a thermostatted silicon oil bath; temperature and torque were continuously recorded during the polymerization. After 30 min, the mixture became transparent, indicating the solubilization of the acid in the glycol. The first stage was conducted at 180 °C under controlled nitrogen flow. In this step, the direct esterification with elimination of water molecules took place (the first phase totally lasted about 4 h). In the second stage, the pressure was gradually reduced to about 0.1 mbar to facilitate the removal of the glycol in excess, and the temperature was risen to 220 °C; the polymerization was carried out until a constant torque value was measured (the second phase totally lasted about 3 h).

The as-synthesized polymer was purified through dissolution in a mixture hexafluoro-2-propanol/chloroform and precipitation in methanol. The purified polymer, in the form of white floccules, was dried at 30 °C under vacuum to constant weight. Thin films of about 150 µm thickness were obtained by compression molding using a Carver press. Purified polymer was melted at 180 °C and kept for 2 min at a pressure of 5 tons/m^2^. Lastly, the film was cooled to RT in press by tap water.

Film thickness was determined by Sample Thickness Tester DM-G (Brugger Feinmechanik GmbH, Munich, Germany). Reported value represents the mean thickness of three experimental tests, each run on 10 different points on the polymer film surface at RT.

### 3.3. Molecular, and Thermal Characterization

Polymer structure was checked by ^1^H-NMR spectroscopy at RT. A Varian Inova 400-MHz (Palo Alto, CA, USA) was used for the measurements.

Molecular weights were determined by gel-permeation chromatography (GPC) at 30 °C with a 1100 HPLC system (Hewlett Packard, Palo Alto, CA, USA) equipped with PLgel 5-μm MiniMIX-C column (Agilent, Milan, Italy). A UV-detector (Hewlett Packard, Palo Alto, CA, USA) was employed as detector. A Hexafluoro-2-propanol/chloroform mixture (5%:95% *v*/*v*) was used as eluent with a 0.3 mL/min flow. A molecular weight calibration curve was obtained with polystyrene standards in the range of molecular weight 800–100,000 g/mol.

TGA was carried out under nitrogen atmosphere by means of a Perkin Elmer TGA7 apparatus (Perkin Elmer, Milan, Italy). Gas flow of 30 mL/min and heating scan of 10 °C/min were used for the analysis.

A Perkin Elmer DSC6 (Perkin Elmer, Milan, Italy) was used for the calorimetric measurements. Weighed samples were encapsulated in aluminum pans and heated to about 40 °C above fusion temperature at a rate of 20 °C/min (first scan), held there for 3 min, and then quenched to −40 °C. Finally, they were reheated from −10 °C to a temperature well above the melting at a heating rate of 20 °C/min (second scan).

To evaluate the presence of a rigid-amorphous phase in PNF and PPF, solvent-treated powders characterized by different crystal/amorphous ratio were prepared by partial melting in DSC to various temperatures in the melting range, quickly cooling inside the instrument below the glass transition temperature and reheating at 20 °C/min.

### 3.4. Water Contact Angle Measurements

Static contact angle measurements were performed on polymer films by using a KSV CAM101 instrument (KSV Instruments, Helsinki, Finland) by recording the side profiles of deionized water drops for image analysis, according to the procedure described by Drelich [33]. Eight drops were observed on different areas for each film, and water contact angles (WCA) were reported as the average value ± standard deviation.

### 3.5. Tensile Tests

The tensile measurements were carried out on rectangular films (5 mm wide and 0.2 mm thick) with a crosshead speed of 10 mm/min by using an Instron 4465 tensile testing machine (Darmstadt, Germany), equipped with a rubber grip and a 100 N load cell. A preload of 1 MPa was applied to each specimen prior to testing. At least five replicates were run and the results are provided as the average ± standard deviation.

### 3.6. Gas Transport Measurements

The determination of the gas barrier behavior was performed by a manometric method, using a Permeance Testing Device, type GDP-C (Brugger Feinmechanik, GmbH, München, Germany), according to ASTM 1434-82 (Standard test Method for Determining Gas Permeability Characteristics of Plastic Film and Sheeting), DIN 53 536 in compliance with ISO/DIS 15 105-1 and according to Gas Permeability Testing Manual (Registergericht München HRB 77020, Brugger Feinmechanik GmbH).

After a preliminary high vacuum desorption of the up and lower analysis chambers, the upper chamber was filled with the gas test, at ambient pressure. A pressure transducer, set in the lower chamber, records continuously the increasing of gas pressure as a function of the time. The gas transmission rate (GTR) was determined considering the increase in pressure in relation to the time and the volume of the device. All the measurements have been carried out at room temperature (23 °C). The operative conditions were: gas stream of 100 cm^3^ min^−1^; 0% RH of gas test, food grade; sample area of 78.5 cm^2^ (standard measurement area). Films were also analyzed at 5°C, 15°C, and 38 °C. Gas transmission measurements were performed at least in triplicate and the mean value is presented. Method A was used for the analysis, as just reported in the literature [34,35] with evacuation of up/lower chambers. Sample temperature was sets by an external thermostat HAAKE-Circulator DC10-K15 type (Thermoscientific, Selangor, Malaysia).

The transport phenomena background followed in the experiment is well described in literature, with a full description of the mathematical equation and interpretation [6,32].

### 3.7. Simulant Liquids

The food contact simulation was performed in accordance with EU Regulations No. 10/2011 on plastic materials and articles intended to come into contact with food [30].

Four solutions were used as food simulants:

- Simulant A, Ethanol 10% (*v*/*v*), 10 days, 40 °C
- Simulant B, Acetic acid 3% (*v*/*v*), 10 days, 40 °C
- Simulant C, Ethanol 20% (*v*/*v*), 10 days, 40 °C
- Simulant D1, Ethanol 50% (*v*/*v*), 10 days, 40 °C

The measurement was made on a totally immersed 12 cm × 12 cm film specimen. 200 mL of simulant was placed into glass flasks (of 400 mL of volume) containing the film sample and the flasks were then covered with caps. Samples were placed in a stove (Universalschrank UF110, Memmert GmbH + Co. KG, Schwabach, Germany). After the assay time was elapsed, the specimens were removed from the flasks, washed with distilled water two times and dried with blotting paper. Before analysis, the films were kept at room temperature, in dry ambient for at least two weeks. The samples were tested in triplicate.

### 3.8. Relative Humidity Solution

According to the procedure reported on the *Gas Permeability Testing Manual (Registergericht München HRB 77020, Brugger Feinmechanik GmbH)*, the analyses were performed at different relative humidity (RH) obtained with several saturated saline solutions. In particular:

- Standard Environment, 23 °C, 85% of RH, with saturated KCl solution;
- Tropical Climate, 38 °C, 90% RH, with saturated KNO_3_ solution;
- 33% RH, 23 °C, with saturated MgCl_2_ solution;
- 57% RH, 23 °C, with saturated NaBr solution;
- 75% RH, 23 °C, with saturated NaCl solution;

The values for the relative humidity for the saline solutions are taken from DIN 53 122 part 2. In the humid part of the top permeation cell was insert a glass-fiber round filter humidified with the desired saturated saline solution. Method C was used, with gas flow blocked onto the test specimen during evacuation. In this manner, the test gas is humidified inside the permeation cell. This method evacuates only the area of the bottom part of the sample. On the top part of the test specimen, with the humidified gas, the normal ambient pressure is applied.

## 4. Conclusions

Poly(neopentyl glycol furanoate), an aromatic polyester derived from renewable resources, has been successful synthesized through melt polycondensation, starting directly from the corresponding dicarboxylic acid. PNF showed superior thermal stability and similar mechanical properties with respect PPF, previously synthesized by us [12].

More interestingly, compared to PEF, PNF displays a reduction of permeability to O_2_ and CO_2_ of 11× and 61×, respectively [5,6] and revealed to be even better than PPF.

In the case of nitrogen gas and especially in the case of carbon dioxide gas, temperature did not show a significant impact on the gas transmission process, and this is an advantage for storage packaging use. On the contrary, oxygen gas was found to be more sensitive to temperature.

Permeability data after contact with food simulants indicate that no significant change occur in the chemical-physical characteristics of the polymer sample, with a very low effect on the permeability behavior.

The permeability increased with increasing RH, indicating a strong interaction with moisture, as presumable, taking into account the polar nature of the furan ring.

The calculated ratios of permeability were different from the ratios of standard polymers reported in literature [22]. This result is important for the selection of the correct headspace gas composition for modified atmosphere packaging, in order to avoid collapsing of the package or in order to choice the correct storage atmosphere condition.

In conclusion, PNF can be considered a very important member of the bio-based polyester family, opening up new possibilities in the sustainable packaging.

## Figures and Tables

**Table 1:** Molecular, thermal, and mechanical characterization data for PNF. * PPF from [12] has been added for sake of comparison.

	PNF	PPF *
**MOLECULAR CHARACTERIZATION**		
M_n_ (g/mol)	34,000	30,000
D	4.0	2.3
WCA (°)	112 ± 2	101 ± 3
**THERMAL CHARACTERIZATION**		
**Thermogravimetric analysis**		
T_onset_ (°C)	364 ± 1	360 ± 1
T_max_ (°C)	395 ± 1	387 ± 1
**Differential scanning calorimetry**		
***1st scan***		
T_m_ (°C)	197 ± 1	168 ± 1
ΔH_m_ (J/g)	30 ± 3	7 ± 4
T_g_ (°C)	73 ± 1	50 ± 1
ΔC_p_ (J/g°C)	0.350 ± 0.002	0.194 ± 0.003
T_cc_ (°C)	146 ± 1	137 ± 1
ΔH_cc_ (J/g)	30 ± 3	7 ± 3
***2st scan***		
T_m_ (°C)	197 ± 1	-
ΔH_m_ (J/g)	27 ± 4	-
T_g_ (°C)	70 ± 1	50 ± 1
ΔC_p_ (J/g°C)	0.304 ± 0.001	0.19 ± 0.001
T_cc_ (°C)	158 ± 1	-
ΔH_cc_ (J/g)	27 ± 3	-
**MECHANICAL CHARACTERIZATION**		
E (MPa)	1648 ± 100	1363 ± 158
σ_B_ (MPa)	45 ± 5	31 ± 3
ε_B_ (%)	4 ± 1	3 ± 1

**Table 2:** Gas transmission rate (GTR) data for PNF, normalized for the thickness film sample (199 micron), at 8 °C, 15 °C, 23 °C and 38 °C, with CO_2_, O_2_ and N_2_ gas test, with the corresponding perm-selectivity ratio and Activation energies (E_GTR_) of the transmission process calculated in the range of 8–38 °C.

T (°C)	N_2_-GTR (cm^3^/m^2^ d bar)	O_2_-GTR (cm^3^/m^2^ d bar)	CO_2_-GTR (cm^3^/m^2^ d bar)	CO_2_/O_2_	CO_2_/N_2_	E_GTRN_2__ (KJ/mol K)	E_GTRO_2__ (KJ/mol K)	E_GTRCO_2__ (KJ/mol K)
8	0.012 ± 1.2 × 10^−4^	0.018 ± 5.0 × 10^−5^	0.018 ± 4.3 × 10^−5^	0.99	1.51	27 (0.8)	38 (1)	29 (0.9)
15	0.019 ± 2.3 × 10^−4^	0.021 ± 1.9 × 10^−4^	0.023 ± 3.2 × 10^−4^	1.10	1.20			
23	0.016 ± 1.6 × 10^−4^	0.032 ± 9.4 × 10^−5^	0.022 ± 3.2 × 10^−4^	0.69	1.38			
38	0.040 ± 3.2 × 10^−4^	0.083 ± 4.3 × 10^−4^	0.062 ± 3.2 × 10^−4^	0.75	1.54			

**Table 3:** Comparison of gas barrier data of PNF with those of other polyesters taken from literature.

Sample	O_2_-GTR cm^3^cm m^−2^ day^−1^ atm^−1^	CO_2_-GTR cm^3^cm m^−2^ day^−1^ atm^−1^	BIF O_2_	BIF CO_2_	References
PNF ^1^	0.0323	0.0223	11	61	This work
PPF ^1^	0.0224	0.0288	16	48	[12]
PPF ^2^	0.0472	n.a.	8	-	[11]
PEF ^3^	0.0702	0.1710	2	8	[5,6]
PET ^3^	0.7480	3.2237	0.5	0.4	[5,6]
PET ^4^	n.a	3.4868	-	0.4	[5,6]
PET ^5^	0.3630	1.37	1	1	[20]
PLA ^6^	1.3349	3.2854	0.3	0.4	[21]

^1^ O_2_ and CO_2_ transmission rate, at 23 °C, amorphous sample; ^2^ at 23 °C, 50% relative humidity, ΔH_m_ = 0.4 J/g; ^3^ at 35 °C; ^4^ at 25 °C; ^5^ at 23 °C, 43% relative humidity; ^6^ at 23 °C, 0% relative humidity.
